# Exploring the evolution of bacterial cellulose precursors and their potential use as cellulose-based building blocks

**DOI:** 10.1038/s41598-024-62462-9

**Published:** 2024-05-21

**Authors:** Francesca Mauro, Brunella Corrado, Vincenza De Gregorio, Elena Lagreca, Concetta Di Natale, Raffaele Vecchione, Paolo Antonio Netti

**Affiliations:** 1 Department of Chemical, Materials and Industrial Production Engineering, University of Naples Federico II, Naples, Italy; 2 Istituto Italiano di Tecnologia, Naples, Italy; 3 Interdisciplinary Research Centre on Biomaterials, University of Naples Federico II, Naples, Italy; 4 Department of Biology, University of Naples Federico II, Naples, Italy

**Keywords:** Bacterial cellulose, Kombucha, Biomaterials, SEM, SHG, Biomaterials, Bioinspired materials, Biomaterials - cells, Tissue engineering, Materials for devices, Structural properties, Biomedical engineering

## Abstract

Natural polymers have found increased use in a wider range of applications due to their less harmful effects. Notably, bacterial cellulose has gained significant consideration due to its exceptional physical and chemical properties and its substantial biocompatibility, which makes it an attractive candidate for several biomedical applications. This study attempts to thoroughly unravel the microstructure of bacterial cellulose precursors, known as bioflocculants, which to date have been poorly characterised, by employing both electron and optical microscopy techniques. Here, starting from bioflocculants from Symbiotic Culture of Bacteria and Yeast (SCOBY), we proved that their microstructural features, such as porosity percentage, cellulose assembly degree, fibres’ density and fraction, change in a spatio-temporal manner during their rising toward the liquid–air interface. Furthermore, our research identified a correlation between electron and optical microscopy parameters, enabling the assessment of bioflocculants' microstructure without necessitating offline sample preparation procedures. The ultimate goal was to determine their potential suitability as a novel cellulose-based building block material with tuneable structural properties. Our investigations substantiate the capability of SCOBY bioflocculants, characterized by distinct microstructures, to successfully assemble within a microfluidic device, thereby generating a cellulose sheet endowed with specific and purposefully designed structural features.

## Introduction

Bacterial cellulose (BC) is an exclusive biomaterial synthesized by several species of bacteria, among which *Gluconacetobacter xylinus* is the most widely studied microorganism able to produce cellulose. BC is obtained by fermentation and appears as a gel-like pellicle floating at the liquid–air interface in common static culture growth conditions^1^. It is assumed that bacteria produce cellulose pellicles to protect themselves from UV radiation and other external environmental agents while preserving moisture^2^. Chemically, it is equivalent to plant cellulose but possesses higher purity due to the absence of lignin, pectin and hemicellulose^3^. Moreover, the unique ultrafine branched structure of BC provides interesting chemical and physical properties such as high water holding capacity, high tensile strength and high crystalline structure^4^, which make BC suitable for a large variety of applications, including food industry, electronics and environmental^5–8^. In addition, it found extensive use in the biomedical area as in wound healing^9,10^, drug delivery^11,12^, tissue engineering and artificial blood vessels^13,14^ due to its non-toxic and biocompatibility properties. Numerous studies reported that bacteria culture conditions can affect the morphology and structure of the BC^15–19^. For example, variations in carbon sources and ethanol concentrations utilized in the cultivation media resulted in alterations in the cellulose membranes generated by diverse *Komagataeibacter* strains, consequently influencing membrane microstructure such as surface porosity, fibre diameter and pores size^20^. Moreover, in recent decades bacterial polysaccharides have seen widespread use in the production of BC-nanocomposites^21^. This involves integrating functional nano-additives and supplementary phases into the bacterial cellulose matrix for diverse applications spanning multiple fields^22^. Additionally, BC finds utilization also in bioprinting processes as an important component of the inks, influencing their fundamental features, including mechanical and thermal properties, as well as printability and biodegradability^23–26^, although in combination with other polymers such as alginate^27^ or gelatin^28^ to make it processable. For example, an alginate sulphate-nanocellulose-based bioink with excellent printing properties has been proposed for cartilage tissue engineering applications^29^. Also, a 3D-bioprinted alginate-GelMA-bacteria nanocellulose scaffold containing RSC96 cells has been fabricated exhibiting remarkable properties, in terms of plasticity and mechanical strength, which provides a favourable microenvironment for cell adhesion and proliferation^30^. Further, a recent study reported the use of a partially acid-hydrolysed Kombucha sheet, as a suitable 3D printed scaffold with good mechanical strength and cytocompatibility^31^. Kombucha tea is a sweet beverage commonly obtained by the fermentation of black tea leaves made by a symbiotic culture of bacteria and yeast (SCOBY)^32^. This beverage has originated in North-Eastern China, but it is nowadays spread worldwide due to its proven health benefits (antioxidant, antimicrobial, anti-inflammatory, and antiaging)^33^. The microbial composition within a SCOBY can vary, typically comprising *Acetobacter* bacterial species, diverse *Saccharomyces* strains, and various other types of yeasts^34^.It has been documented that the symbiotic interaction between acetic acid bacteria (AAB) and yeasts contributes to increase cellulose yield production. This enhancement arises from the synergistic metabolic activities of these microbes^35^. Furthermore, studies reported that Kombucha fermentation conditions, such as the concentration of culturing media components^36^ or vessel geometry (i.e. availability of surface area for BC production)^37^ affect the chemical-physical properties of the produced BC. Additionally, fungi from Kombucha tea SCOBY were demonstrated to produce effective bioflocculants in well-optimized culture conditions^38^. Bioflocculants, from now on Bioflocs, are extracellular biopolymers produced by various microorganisms, including actinomycetes, fungi, algae, and bacteria, known to induce biofilm formation and microbic agglomeration^39^*.* Due to their biodegradability and biocompatibility, bioflocs obtained high attention in wastewater treatment applications, as an alternative solution to chemical flocculants which can cause environmental and human health risks^40,41^. Although bioflocs have been proven to have antioxidant, anti-inflammatory and antibacterial effect^42^, little is known regarding their mechanism of maturation and assembly which has an impact on the final microstructure and thus on the applications. Therefore, with the increase in the design and development of advanced cellulose-based biomaterials^43^ and with the aim to improve awareness on bioflocs maturation mechanism to get a control on the microstructure of the final material, we characterized SCOBY bioflocs produced during static fermentation conditions and assessed the ability to use them as building blocks for the realization of a novel material with controlled structural properties. In detail, scanning electron microscopy (SEM) and second harmonic generation (SHG) were used to evaluate structural parameters of SCOBY bioflocs collected in different spatial and temporal points of the culture system, in terms of cellulose production and network organization and then accordingly classified in three different categories by means of a colorimetric heat map. The chemical-physical properties were then corroborated by Infrared spectroscopy (IR). Afterwards, bioflocs with different features have been assessed as building blocks by placing them in a constrained environment under continuous culture medium flux. A microfluidic chamber was chosen for its ability to guarantee a controlled environment and optical accessibility^44^. Here, a final cellulose-based material was obtained by assembling bioflocs within the chamber of a PDMS-based microfluidic device keeping their starting structural features. This investigation represents a notable advancement for the comprehension of the structural features of SCOBY bioflocs nanocellulose and its usage in the fabrication of cellulose materials with tuneable properties.

## Results

### Biofloc morphological and chemical characterization

**Figure 1:**
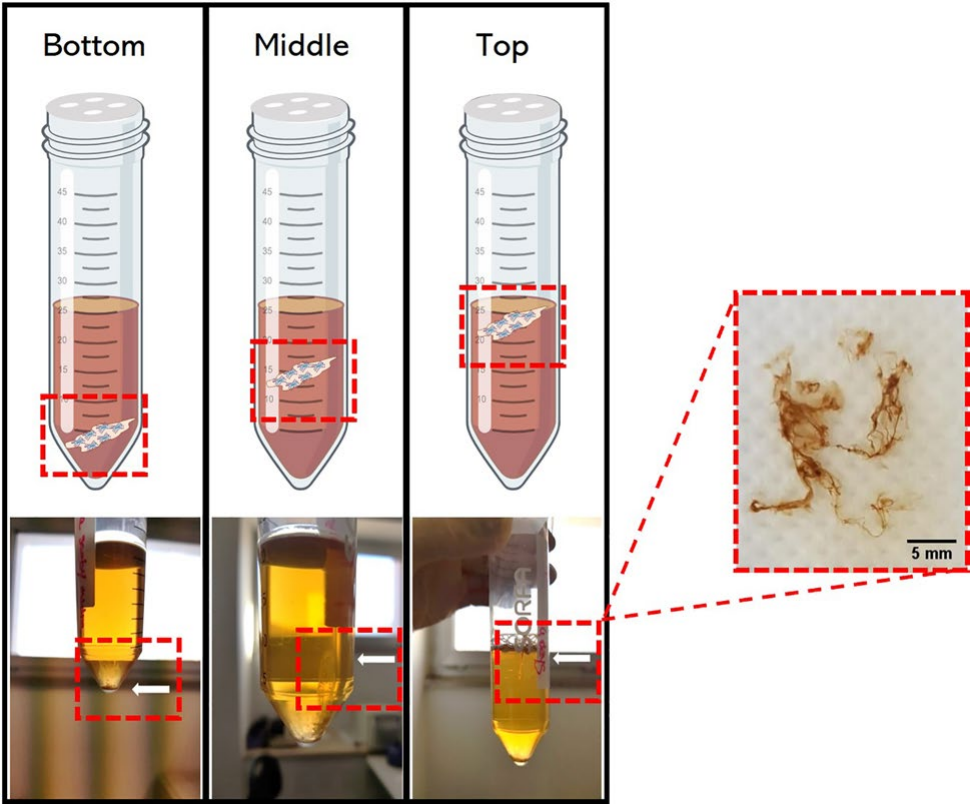
SCOBY bioflocs culture under static conditions. A schematic illustration, accompanied by corresponding images, represents the SCOBY bioflocs harvested from distinct vertical positions–bottom, middle, and top–within the liquid medium during static culture growth. The images of bioflocs are presented in the red-dashed inset for visual reference.

The characterization of SCOBY bioflocs was conducted by selectively harvesting specimens from distinct vertical positions within the culture broth. Specifically, bioflocs were collected from the bottom (B), the middle (M) and the top (T) of the liquid medium column within the tube, as depicted in the schematic representation presented in Fig. 1. Moreover, optical images obtained in brightfield of the bioflocs at each vertical level revealed their composition, comprising agglomerations of bacteria and yeasts embedded within a matrix network (see Supplementary Fig. S1).

Furthermore, their evolution over time was evaluated by IR every 24 h after inoculation from the SCOBY mother culture, until 72 h when the final cellulose sheet formed at the liquid–air interface. Indeed, the characterization of BC using IR spectra is a well-established technique for analysing its structure. In agreement with the literature, IR revealed the characteristic spectra of BC at all time points and vertical positions, stating the presence of a cellulosic component within the bioflocs (see Supplementary Fig. S2). Specifically, the wide spectrum emerged at 3350–3340 cm^−1^, representing the distinctive absorption peaks of BC attributed to the stretching vibration of intramolecular hydrogen bonds. The less intense absorption bands observed at 2900–2800 cm^−1^ represent the C–H stretching vibration. The absorption signals at 1730 cm^−1^ indicated the stretching vibration of the C=O bond, while the peaks at 1630 cm^−1^ were related to the bending of the OH groups, which could be attributed to the absorption of water molecules into cellulose fibres. Moreover, the absorbance bands at 1400 cm^−1^, associated with symmetric CH_2_ bending vibration, were connected to the degree of crystallinity. Additionally, it is confirmed the presence of peaks observed at 1040 cm^−1^, corresponding to the vibration of the pyranose ring –C–O–C, and the peak at 890 cm^−1^, indicating the presence of β-glycosidic bonds^45,46^. Lastly, the potential presence of residual culture medium and/or microorganisms due to not purified BC did not interfere with our analyses, as the typical peaks were clearly discernible. Indeed, it is reported that the purification process does not change the IR spectra of BC compared to the unpurified control^47^.

### SEM and SHG ultrastructural analyses

**Figure 2:**
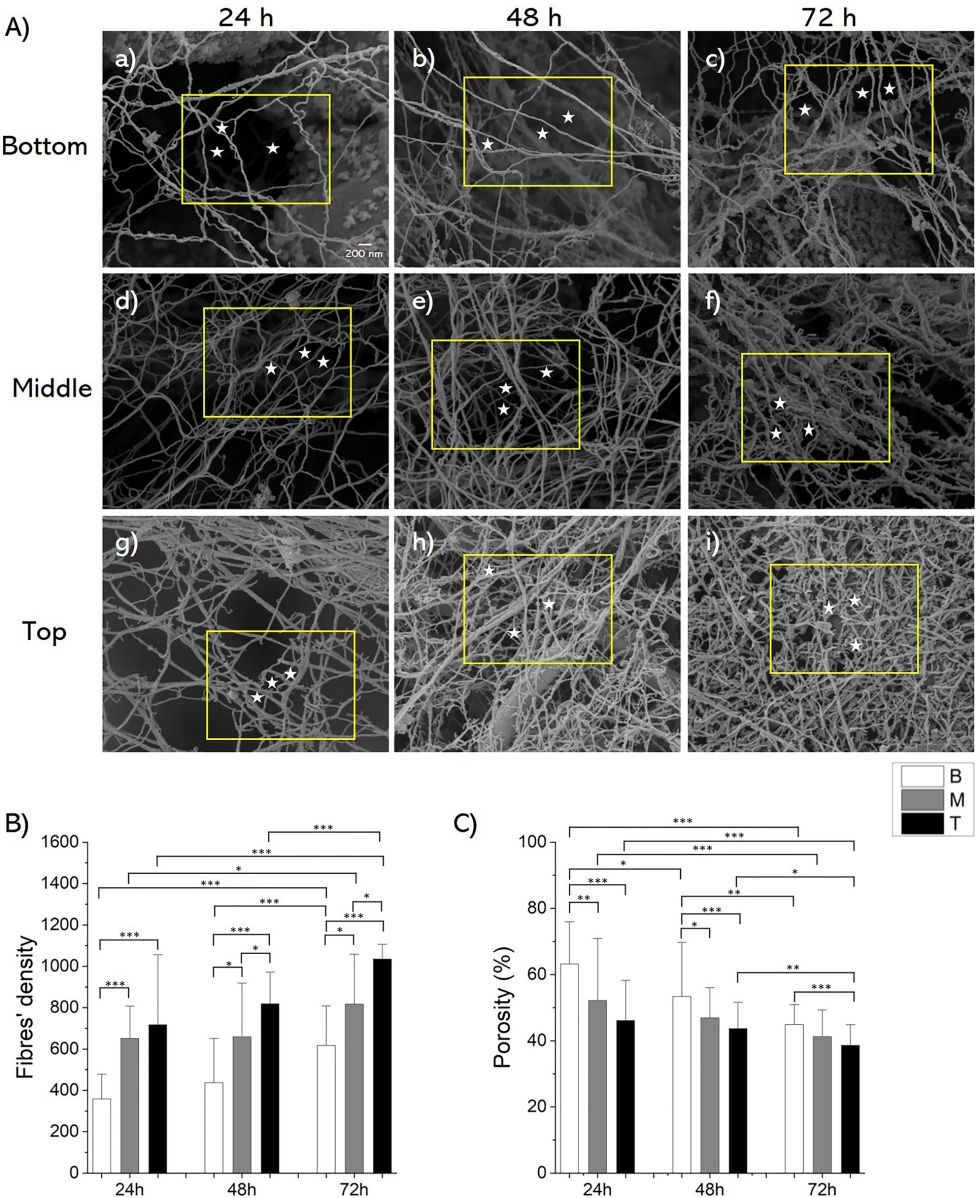
Ultrastructure characterization of SCOBY bioflocs. (**A**) SEM images representing B (a, b, c), M (d, e, f) and T (g, h, i) bioflocs at 24 h (a, d, g), 48 h (b, e, h), and 72 h (c, f, i). White stars in the yellow outlined area indicate the presence of pores. (**B**) Quantitative analysis of the fibres’ density within the cellulose network of the bioflocs. (**C**) Evaluation of the porosity percentage in the biofloc cellulose network. Results were presented as mean values and standard deviation. Statistical significance is assessed through the Kruskal–Wallis test (***p < 0.005, **p < 0.01, *p < 0.05, not significant when not shown, n ≥ 12 for condition).

To comprehensively understand the morphological features of SCOBY bioflocs, they were harvested from the bottom (B), middle (M), and top (T) regions at 24 h, 48 h, and 72 h from the culture broth and subjected to SEM and SHG ultrastructural characterization. SEM images of the bioflocs, presented in Fig. 2A, corroborated the presence of a cellulose network within the bioflocs. Moreover, measurements of fibres’ diameter, fibres’ density and matrix porosity percentage were conducted, characterizing the spatial and temporal aspects of the biofloc structure. Results indicated a consistent mean fibre diameter of 60 nm, derived from distribution Gaussian fitting, exhibiting no significant variation among different biofloc samples (see Supplementary Fig. S3). However, the fibres’ density exhibited a spatial–temporal growth pattern, with T bioflocs consistently demonstrating higher fibres’ density compared to B at each evaluated time point. Further, a significant difference was observed between M and T bioflocs at 48 h and 72 h (*p* < 0.05). Additionally, fibres’ density in both B and T bioflocs exhibited an increasing trend over time, for each evaluated time point, whereas M bioflocs showed a significant increase only between 24 and 48 h (*p* < 0.05) (Fig. 2B). Conversely, the porosity percentage displayed an inverse spatial evolution. Initially, at 24 h and 48 h, M bioflocs presented lower porosity compared to B bioflocs. Subsequently, at 48 h and 72 h, porosity was significantly different only between B and T bioflocs, with T bioflocs having lower values (*p* < 0.005). Furthermore, B bioflocs at 24 h displayed a higher porosity percentage compared to B bioflocs at 48 h and 72 h. In contrast, the porosity of M and T bioflocs at 24 h was similar to that exhibited at 48 h, but significantly different from that at 72 h (*p* < 0.005) (Fig. 2C). Finally, analyses of mean pore area, number of pores, and fibre intersection density within the cellulose network of bioflocs were provided, revealing a spatio-temporal decrease in mean pore area and an increase in both the number of pores and intersection density, in agreement with the observed porosity reduction and cellulose content increase (see Supplementary Fig. S4). A cellulose fibres densification, along with a reduction of porosity, can be also visually detected in the SEM images in Fig. 2A, where pores are indicated by white stars.

**Figure 3:**
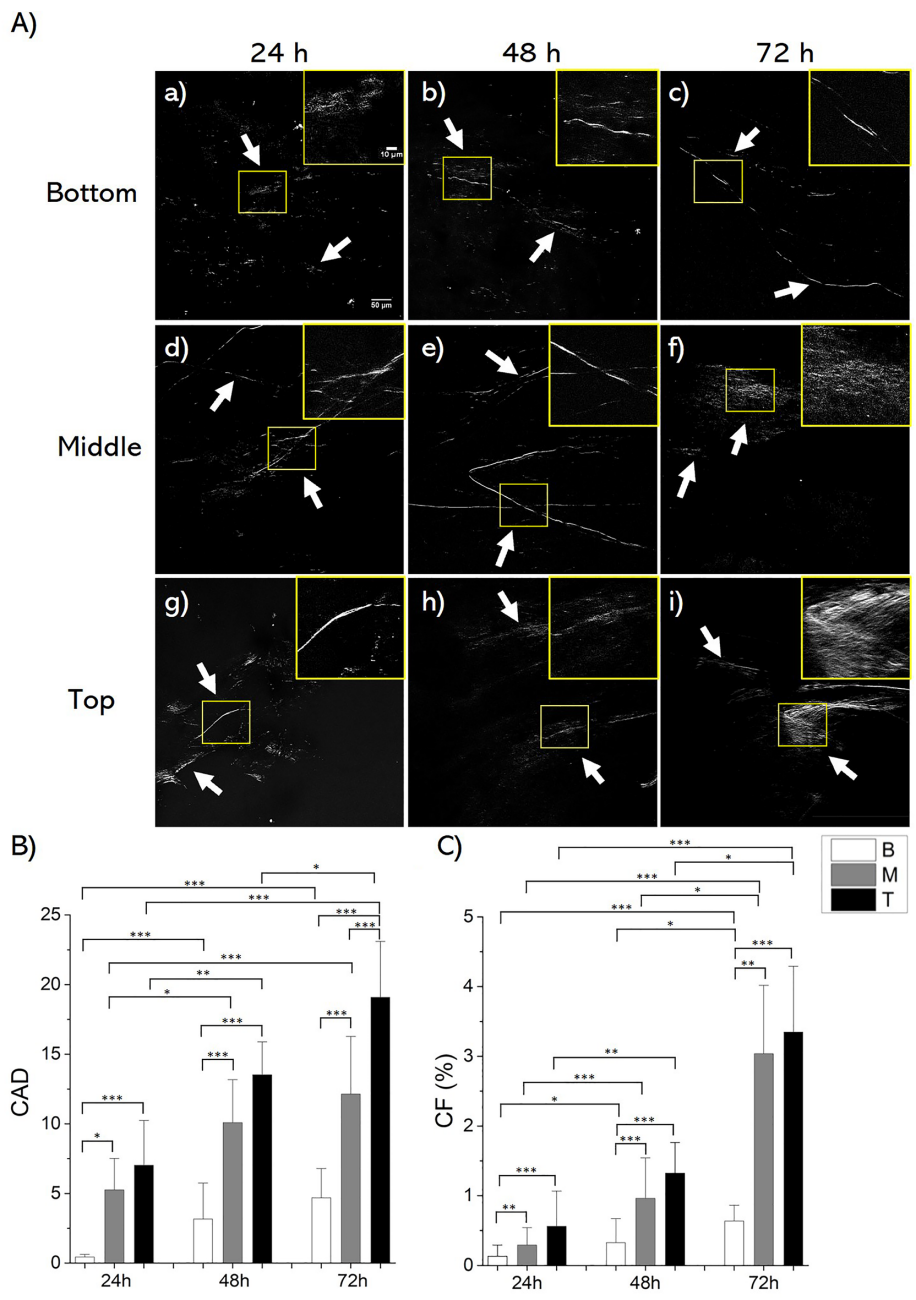
SHG analysis of SCOBY bioflocs structure. SHG images illustrating B (a, b, c), M (d, e, f) and T (g, h, i) bioflocs at 24 h (a, d, g), 48 h (b, e, h), and 72 h (c, f, i). White arrows indicate cellulose fibres generating SHG signal. (**A**) Magnified view of the area outlined in yellow is shown in the upper right corner. Results of (**B**) CAD and (**C**) CF. Results were presented as mean values and standard deviation. Statistical significance is assessed through the Kruskal–Wallis test (***p < 0.005, **p < 0.01, *p < 0.05, not significant when not shown, n ≥ 10 for condition).

Additionally, Cellulose Assembly Degree (CAD) and Cellulose Fraction (CF) were quantified from SHG images of bioflocs (Fig. 3A), providing insights into cellulose maturation and quantity, respectively. Indeed, SHG can detect the non-centrosymmetric and birefringent cellulose nanofibers present within the bioflocs. In particular, CAD values were derived from SHG signal intensity levels, revealing an ascending trend from B to T bioflocs at each examined time point. Additionally, CAD values exhibited an increase over the growth period for all the bioflocs collected at the different positions (*p* < 0.05, *p* < 0.005) (Fig. 3B). Furthermore, CF values displayed a parallel spatial and temporal evolution, indicating an elevation in T and M bioflocs compared to B bioflocs, and a progressive increase during their growth period (Fig. 3C). Indeed, at earlier time points and lower positions, cellulose fibres, indicated by the white arrows in Fig. 3A, appeared sparse and fragmented, while at later time points and higher positions they appeared denser and more compact.

### Bioflocs classification and SEM-SHG correlation

**Figure 4:**
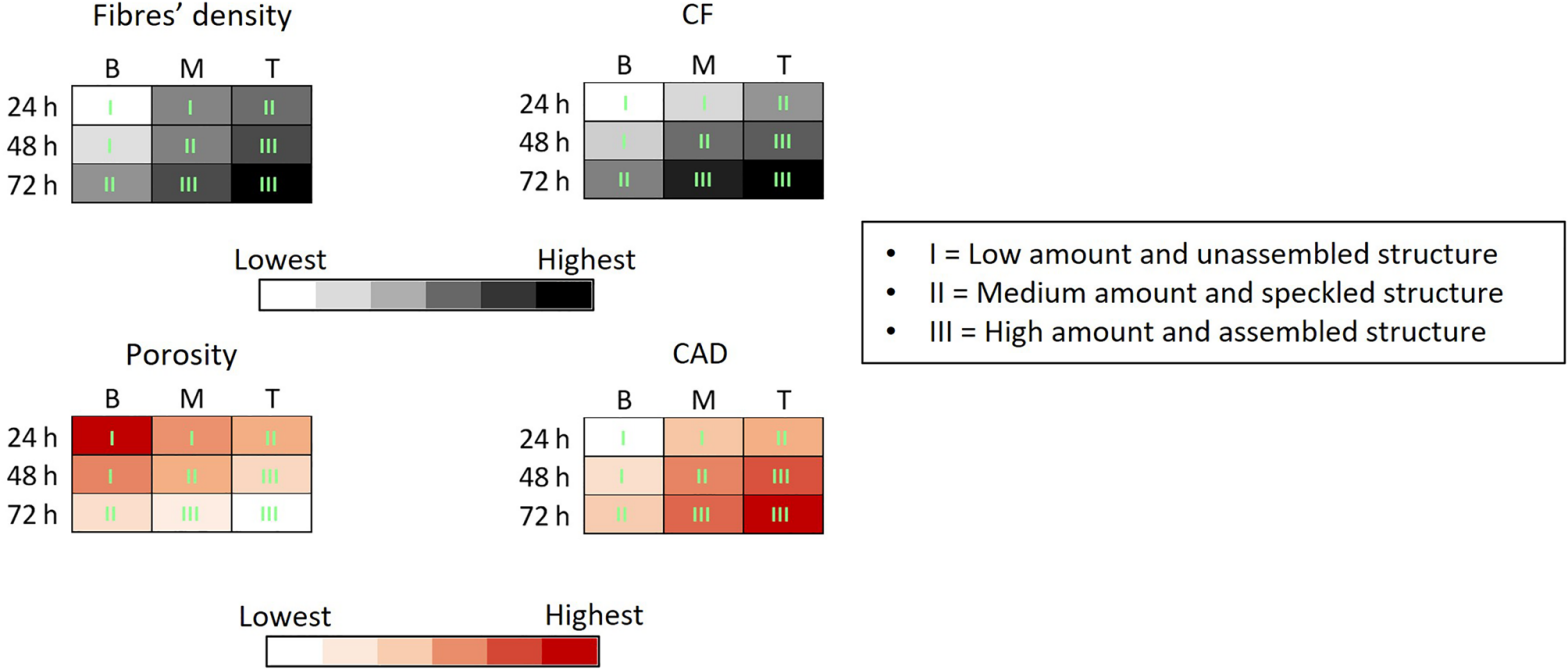
Colorimetric maps of SCOBY bioflocs for their structural features detection. Colorimetric maps were generated for the identification of SCOBY biofloc networks based on the values of cellulose fibre’s density, CF, porosity, and CAD.

Based on the structural parameters derived from both SEM and SHG analyses, SCOBY bioflocs were categorized into distinct groups. Colorimetric maps representing B, M, and T bioflocs at different culture times were generated to qualitatively discern each type of bacterial biofloc. This was achieved through a colour code spanning from low to high values of fibres’ density/CF and porosity/CAD, providing insights into the cellulose content within the bioflocs and the structure of the cellulose network, respectively (Fig. 4). Consequently, bioflocs were classified into three distinct classes based on cellulose amount (low, medium, high) and the type of structure (unassembled, speckled, assembled). This classification could be a useful tool for easy and qualitative identification of SCOBY bioflocs microstructure. Furthermore, we observed a correlation among structural parameters of bioflocs as measured from SEM and SHG images. Specifically, fibres’ density and CF exhibited a congruent spatial and temporal progression, indicating a positive correlation. Conversely, porosity and CAD displayed a negative correlation, with porosity diminishing as CAD increased (see Supplementary Fig. S5). This finding can serve as a potent method to evaluate cellulose microstructural characteristics without necessitating pre-processing procedures, such as those typically employed in SEM, or the application of staining dyes.

### Bioflocs assembly within a confined microfluidic-based device

**Figure 5:**
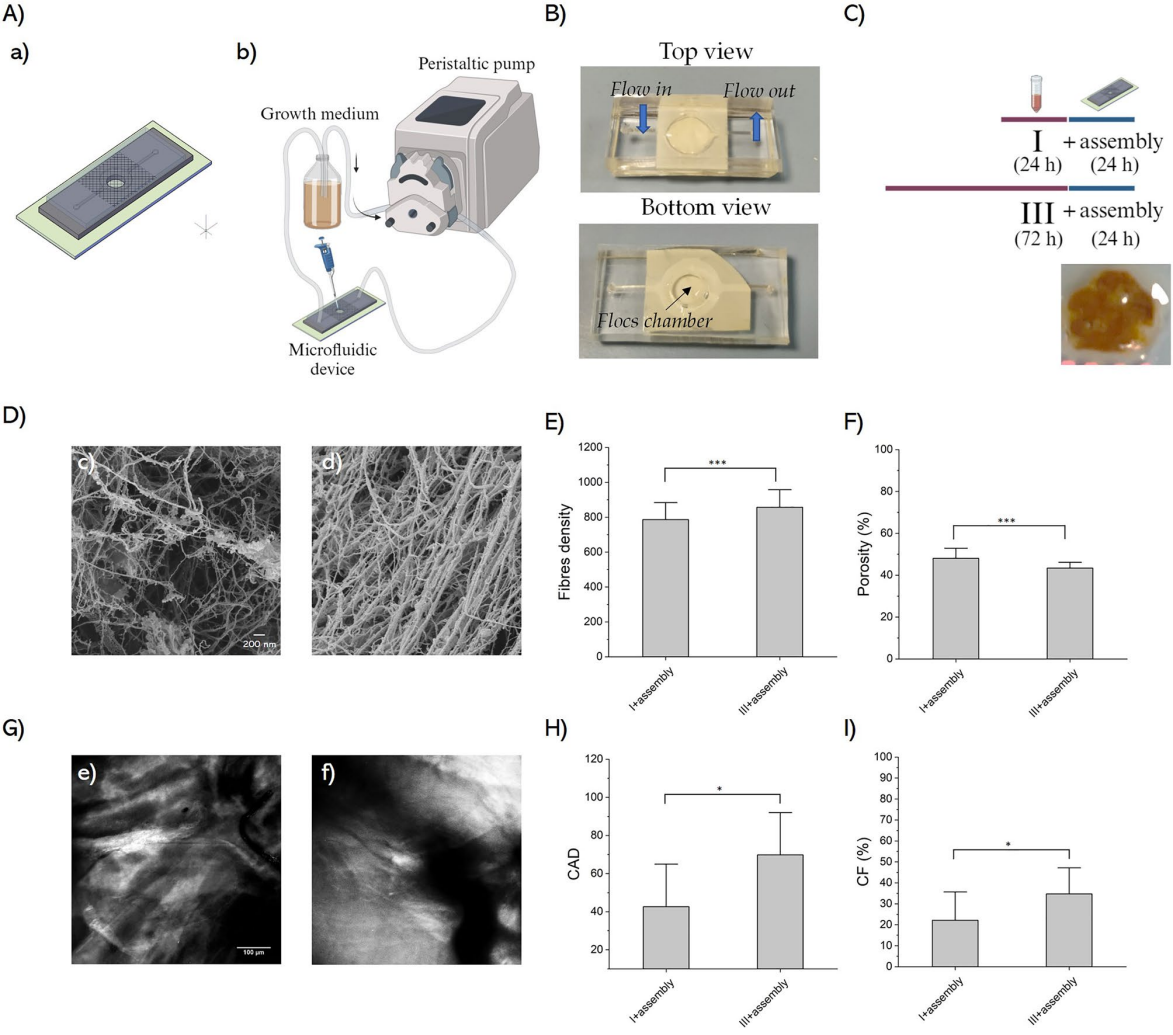
Bioflocs building blocks assembly within the microfluidic device. (**A**) Assembly set-up. CAD of the microfluidic device for biofloc assembly (a). Graphical representation illustrating the biofloc assembly setup (b). (**B**) Top and bottom view images of the microfluidic device and the assembly chamber. (**C**) Schematic depiction of the experimental procedure for biofloc assembly within the microfluidic device. In the lower right corner, an image representing the cellulose sheet assembled within the microfluidic device is presented. (**D**) SEM images illustrating cellulose sheets from assembled class I (c) and class III (d) bioflocs within the microfluidic device after 24 h of culture. Results of (**E**) fibres’ density and (**F**) porosity percentage. Statistical significance is assessed through a Two-sample t-test (***p < 0.005, not significant when not shown, n ≥ 14 for condition). (**G**) SHG images of cellulose sheets from assembled class I (e) and class III (f) bioflocs. Results of (**H**) CAD and (**I**) CF. Statistical significance is assessed through a Two-sample t-test (*p < 0.05, not significant when not shown, n ≥ 10 for condition). Results were presented as mean values and standard deviation.

The microfluidic device was designed to facilitate the cultivation and assembly of cellulose bioflocs. Following the introduction of bioflocs into the circular chamber via pipetting, the microfluidic device was temporarily sealed with metal clamps. Subsequently, the chip was connected to a peristaltic pump and subjected to perfusion at a defined, constant flow rate (Fig. 5A). Within the chip, the convective flow of media was restricted to the upper media chamber. Nevertheless, the porous membrane separating the lower culture chamber and the upper media chamber allowed for the diffusion of dissolved oxygen and nutrients from the perfusing media to the bioflocs, as well as metabolites and secreted factors from the bioflocs into the media. Top and bottom views of the device are presented in Fig. 5B. To investigate the assembly of SCOBY bioflocs, specimens with the extreme structural features (class I and III) were collected from the culture medium and then allowed to assemble within the microfluidic chip for an additional 24 h-sufficient time for bioflocs to form a unified cellulose sheet (Fig. 5C). Subsequently, the assembled cellulose sheets were collected and subjected to SEM and SHG analyses to assess their ultrastructural characteristics. SEM image analyses (Fig. 5D) revealed that the cellulose sheet obtained from class III bioflocs exhibited a higher fibres’ density and lower porosity percentage after 24 h of assembly within the microfluidic device compared to that produced by class I bioflocs (Fig. 5E,F). Additionally, it displayed a lower mean pore area, a higher number of pores, and greater fibres intersection density (see Supplementary Fig. S6). Further, analyses of SHG signals (Fig. 5G) reported higher CAD and CF in the cellulose sheet obtained from class III bioflocs compared to that from class I bioflocs (Fig. 5H,I). Moreover, additional evaluations were conducted to further confirm our findings by staining the cellulose sheet with calcofluor white, a non-specific fluorochrome known for its affinity to cellulose and chitin within cell walls^48^. Analyses of cellulose fraction and mean fluorescence intensity from the fluorescent images revealed elevated values in the cellulose sheet derived from class III bioflocs (see Supplementary Fig. S7), thereby corroborating the findings obtained through SEM and SHG analyses. All these pieces of evidence highlight that this bioflocs assembly approach enables the control of the microstructural features of the final material by starting with bioflocs building blocks with different characteristics.

## Discussion

BC has gained great attention in recent years as a promising biological material for fabricating 3D cell culture platforms due to their structural analogies to the fibrous morphology of the ECM, along with its excellent biocompatibility and water retention capability^49^, which was proven to be a relevant parameter to consider in biological applications^50^. Some microorganisms can intracellularly synthesize a biopolymer and expel it through their cellular wall. In our case, we use the microorganisms present in the SCOBY which can produce cellulose. The resulting biopolymer has a tendency to clump together, forming small cellulose precursors known as bioflocs. These bioflocs are environmentally biodegradable and biocompatible, commonly used in wastewater treatment as solid particle retainers. This dual functionality underscores the versatility and potential impact of BC in both biological and environmental contexts^51^. However, to our knowledge, there have been no reports presenting a comprehensive spatio-temporal structural analysis of SCOBY bioflocs or regarding their possible applications in different fields, which may be valuable for the exploitation of bioflocs with various features serving as building blocks. Indeed, a recent study has focused on a systematic characterization of mycelium morphology throughout its growth, which has revealed of great relevance for the comprehension of its structural properties^52^. Therefore, the goal of this work was to characterize the morphological, chemical, and physical properties of bioflocs obtained from Kombucha tea SCOBY fermentation in order to use them as building block materials with tuneable microstructural features. We selected SCOBY due to its robustness against potential contaminations and its ability to adapt to various environmental conditions, including nutrients, which can even include vegetable waste, rendering it highly appealing from a practical standpoint. Additionally, we introduced a methodology applicable not only to pure bacterial cultures but also to other microorganism mixtures like those found within SCOBY. Therefore, in light of SCOBY's well-established capacity for biofloc production^38^, our investigation was specifically oriented towards the bioflocs generated within the SCOBY culture. In the early stages (24-h post-inoculation) a notable accumulation of bioflocs was observed predominantly at the bottom of the tube with few flocs detected at the liquid–air interface. By 72 h, the bioflocs were distributed throughout the entire volume, resulting in the formation of a dense, cohesive and transparent cellulose pellicle at the air–liquid interface. To investigate the spatio-temporal and structural organization, SCOBY bioflocs were collected at different vertical positions within the liquid broth column (Bottom, Medium and Top) at diverse growth timepoints after inoculation from the mother culture (24 h, 48 h and 72 h). Our findings revealed a consistent clustering morphology across all bioflocs types, constituting microorganisms entwined within an extracellular matrix. IR analysis confirmed the presence of bacterial cellulose, consistent with previous works^38,53^. To further characterize the product, we investigated the structural characteristics of SCOBY bioflocs matrix using SEM and SHG measurements. Notably, cellulose, as well as collagen, exhibits a non-centrosymmetric helical structure, generating SHG signals^54–56^. The dual analytical approach (SEM and SHG) allowed for a comprehensive examination of the bioflocs' fibres’ density/porosity and CF/CAD, respectively. Concerning SEM ultrastructural analyses, the results revealed a higher amount of cellulose fibres within bioflocs positioned at the top (T) compared to those at the middle (M) and bottom positions (B), all exhibiting a mean diameter of 60 nm, which is characteristic of cellulose produced through conventional static culture methods^57^.This is in agreement with multiple studies noting a contrasting spatial arrangement of nanofibrils between the bottom and top surfaces of cellulose pellicles. Specifically, the surface exposed to air exhibited a denser structure, while the surface in contact with the growth medium displayed more porous regions^58–60^. This variation is ascribed to differences in oxygen distribution, resulting in varying levels of active bacteria^61^. Furthermore, we observed a progressive increase in cellulose fibres content over the culture duration for each biofloc at different vertical positions within the tube. Conversely, porosity showed an inverse spatial and temporal evolution. In fact, it is reported that extended fermentation periods yield increased fibril quantities, leading to the formation of a denser structure with decreased porosity^62^. Substantially, as the bioflocs produced cellulose, their matrix network thickened, subsequently reducing its porosity. This phenomenon could potentially impact the overall density of the bioflocs. Analyses of the bioflocs network ultrastructure (mean pore area, pores number and fibres intersection density) further support this hypothesis. Moreover, CF and CAD measurements, usually performed on collagen^63^ and adapted here to cellulose, were carried out on SHG images of bioflocs. The outcome of the resulting SHG analysis showed remarkable similarity with the observation of the bioflocs ultrastructure. The observed increase in CF from B to T bioflocs, along with its temporal progression, suggests a dynamic relationship between CF and biofloc development. Concurrently, the rise in CAD values on both spatial and temporal biofloc growth indicates a cellulose remodelling and maturation. Indeed, elevated CAD values have been recently correlated with increased collagen fraction and improved extracellular matrix (ECM) remodelling in an in-vitro model of the human intestine^64^. The diverse microstructure at different vertical positions can be justified by the fact that upon biofloc migration induced by lower density, as compared to water, bioflocs encounter different concentrations of oxygen^65^. Indeed, it has been highlighted that, during the initial growth phase, bacteria engage in prolific cellulose production by using dissolved oxygen within the liquid medium. Following the depletion of dissolved oxygen, cellulose generation becomes sustained only in bacterial cells situated near the liquid surface^66^. We proved that, as bioflocs ascend towards the liquid–air interface, there is an increase in cellulose content accompanied by a reduction in the matrix's porosity. Interestingly, our study revealed a correlation between ultrastructural and optical measurements. Of note, fibres’ density and CF increased accordingly, while porosity decreased as CAD increased. This correlation suggests that SHG, being label-free and avoiding sample processing required for electron microscopy, could be used as a valuable tool for continuous in-line assessment of bacterial floc ultrastructure. Actually, adding calcofluor white dye to the medium may represent a further optical method for real time BC ultrastructure monitoring with no need for two-photon excitation confocal microscope.  Consistently with parameter values obtained from both SEM and SHG analyses, we generated a colorimetric map to classify bioflocs based on their cellulose amount (low, medium and high) and structural type (unassembled, speckled and assembled). The resulting classification enabled a rapid and straightforward qualitative detection of bioflocs micro and nanostructure for their use as building blocks of matrices/scaffolds. To leverage these findings for final applications of the bioflocs, we exploited both time and space to segregate bioflocs with varying characteristics. This enabled us to construct pure cellulose materials with diverse predictable and controllable features. Thereby, we hypothesized the possibility of assembling bioflocs just by using cellulose produced by living bacteria embedded in the bioflocs and that the properties of the final assembled material could be controlled and finely tuned by selectively choosing SCOBY bioflocs with a specific cellulose amount/structure. To this aim, we firstly investigated the ability of isolated bioflocs collected from static cultures to assemble within a confined microfluidic-based device composed of a single chamber, where bioflocs were placed and allowed to grow and aggregate under dynamic culture conditions. Microfluidic devices not only provide a confined environment but also allows nutrient diffusion and oxygen supply which are crucial for bacterial metabolism and cellulose synthesis^67,68^. Remarkably, during the assembly process within the microfluidic device, the bioflocs coalesce to form a cohesive cellulose block. This phenomenon is likely due to the ability of bacterial cells within the bioflocs to generate new cellulose fibres, facilitating the formation of a distinctive interconnected matrix, thus suggesting the capability of SCOBY bioflocs to be exploited as building blocks for the self-assembly of functional biomaterials^69^. As an example, BC spheroids produced by *K. rhaeticus* were used as building blocks for the regeneration of cellulose-based engineered living materials (ELMs). More specifically, they offer a method for integrating small pieces of cellulose, obtained from agitated culture conditions, and synthetic materials into a unified composite structure^70^. Herein, the use of bioflocs with distinct initial structures (class I and III) led to the production of a cellulose sheet characterized by varying cellulose content and maturation levels. Of note, relative structural variations observed in the final cellulose sheet inside the microfluidic chamber mirrored those found in the initial bioflocs, analogously to a sintering process, where particles fusion under heat and/or pressure results in a cohesive solid piece, whose characteristics are dictated by those of the raw material^71^. In this analogy, SCOBY bioflocs have been revealed to serve as the building blocks that generate a unified cellulose sheet, whose physical features are influenced by those of the original bioflocs. Differently from pure chemo-physical sintering, in the case of BC, being a living system, the densification mechanism originates from the synthesis of new fibres produced by active bacteria under conventional culture conditions. These preliminary findings highlight the potential of SCOBY bioflocs for producing a cellulose-based building block material with specific structural properties able to dictate the architecture of the final matrix/scaffolds. Nevertheless, further experiments must be conducted in order to definitely establish the suitability of the bioflocs in various applications. For instance, the purification of bioflocs from bacterial cells and their incorporation with bioactive signals warrant investigation to develop a comprehensive biocompatible matrix. This matrix would be capable of sustaining cell viability and regulating specific cellular activities, making it suitable for tissue engineering applications, among others.

## Conclusion

This study represents a significant advancement in the understanding of the BC structure derived from SCOBY bioflocs, revealing crucial insights into the spatio-temporal dynamics governing cellulose production and maturation as bioflocs progress toward the liquid–air interface in static culture conditions. Notably, our observations revealed a distinct increase in fibre’s density, cellulose fraction and assembly degree, accompanied by a simultaneous reduction of porosity, highlighting the intricate spatial and temporal dynamics of these processes. The correlation between electronic and optical characterization holds promise for possible real-time monitoring of the BC maturation. Furthermore, in this work, we successfully assembled SCOBY bioflocs within a confined microfluidic-based device, generating a cohesive cellulose sheet, that retains the key morphological features of the initial bioflocs. These findings offer valuable preliminary insights into the potential application of SCOBY bioflocs as a promising viable building block material for the fabrication of biomaterials with tuneable microstructures and shapes for potential applications in the biomedical fields and beyond.

## Materials and methods

### SCOBY bioflocs culture and extraction:

To cultivate Kombucha SCOBY (KEFIRA), a tea broth is prepared using the following method, as described in our previous work^53^: 860 mL of deionized water (dH2O) is boiled, followed by the addition of glucose (140 g/L). Then, 10 sachets (20 g) of black tea are steeped in the boiled water for 10 min. Afterwards, the tea bags are removed, and the sweetened tea is cooled to room temperature. Next, apple vinegar (140 mL/L) is added to the mixture. The medium is sterilized by autoclaving at 121 °C for 15 min. For each experiment (n = 3), a piece of SCOBY (1 cm × 1 cm) is aseptically introduced into the liquid broth (20 mL within a 50 mL tube) and cultured under static conditions for 3 days as a starter. For biofloc culture, a 1 ml aliquot of the starter fermented SCOBY suspension, after having been gently agitated, is inoculated into the culture broth at a concentration of 5% (1 mL/20 mL). Biofloc production take place under controlled static fermentation conditions in a dark incubator with a humidified atmosphere (≥ 80%) and constant temperature at 30 °C. SCOBY bioflocs are collected from the culture broth, under sterile conditions, by using a micropipette and washed three times with MilliQ before characterization. Spatio-temporal microstructure studies are conducted by selectively harvesting specimens from distinct vertical positions within the culture broth (bottom, middle and top) and different growth-timepoints (24 h, 48 h and 72 h). To ensure the reproducibility and reliability of our findings, different SCOBY batches and teabags substrates are employed to replicate the experiments.

### Infrared spectroscopy analyses

The chemical structure of SCOBY bioflocs is assessed by IR, as described in our previous work^53^. Briefly, after air drying, the sample and control spectra are recorded in the range of 500–4000 cm^−1^ in absorption or transmission modes (64 scans, 4 cm^−1^ resolution) (Thermo Fisher Scientific Instruments, Nicolet 6700, Waltham, MA, United States) and subjected to ATR correction, smoothing, and baseline to be normalized.

### SEM

To gain insight into ultrastructural analysis, the SCOBY bioflocs undergo a series of preparatory steps. They are firstly fixed with 4% paraformaldehyde solution, followed by fixation with 2.5% glutaraldehyde in 0.1 M sodium cacodylate for 1 h at room temperature. Subsequently, they undergo three washes of 0.1 M sodium cacodylate for 10 min each at room temperature. They are then buffered with 1% osmium tetroxide (OsO4) in 0.1 M sodium cacodylate for 1 h at 4 °C, followed by three washes with 0.1 M sodium cacodylate buffer solution. Dehydration is carried out on the samples using ethanol concentrations of 30%, 50%, 70% and 95% for 60 min at 4 °C, followed by three rounds of 100% ethanol for 60 min at room temperature thrice. A critical point dryer (EM CPD300) is used for complete dehydration, after which the samples are gold-coated and subjected to scanning electron microscopy (SEM) (Ultraplus Zeiss). SEM images (1024 × 768 pixels) are obtained and subsequently analysed by using the DiameterJ plugin of ImageJ^72^. First of all, the images are segmented into binary forms using algorithms provided by “DiameterJ Segment” to convert the image. The segmented images are then processed by DiameterJ to measure parameters such as fibres’ diameter and density, porosity percentage, mean pore area, intersection density and the number of pores of the bioflocs cellulose network. All these parameters are determined by the software. The fibres’ density is derived from the histogram of fibre diameters, whereby the frequency sum across the diameter spectrum is divided by the total area of the image, giving an indirect measure of the cellulose content. Bacteria and metabolites are excluded from the analyses to avoid potential errors in the measurements.

### SHG

SCOBY bioflocs are structurally characterized by a confocal microscope (Leica Microsystems, Germany) using a Second-Harmonic Generation (SHG) modality. In detail, all the samples are imaged by two-photon excited fluorescence at the emission microscope (Leica TCS SP5 II coupled with a multiphoton microscope where the NIR femtosecond laser beam is derived from a tuneable compact mode-locked titanium: sapphire laser-Chamaleon Compact OPO-Vis, Coherent). We use an excitation wavelength of k_ex_ = 840 nm (two photons) and collect the signal at an emission wavelength of k_em_ = 420 ± 5 nm. Bioflocs are collected from the liquid broth, washed three times with MilliQ and directly laid on a clean specimen glass slide. Z-stacks are acquired (25×) at 840 nm in back-reflection mode. The Cellulose Assembly Degree (CAD) is evaluated by analyzing the intensity of the SHG signal. Precisely, the signals are subjected to noise subtraction and then the average intensity is evaluated as described by Eq. (1):

1 $$CAD \cong I=\frac{{\sum }_{i=1}^{255}{I}_{i}{p}_{i}}{\sum_{i=1}^{255}pi}$$

where I is the average intensity, I_i_ is the grey-scale SHG signal intensity of the pixel p_i_, while the index i = xi, yi runs in the grey value interval from 1 to 255. The intensity I of the cellulose network is known to be proportional to the degree of assembly of the newly synthesized cellulose. The difference in brightness of SHG signals corresponds to different assembly (or maturation) of the cellulose matrix. For the quantification of the CF, the cellulose portion in selected regions of interest (ROI) is analyzed. The cellulose portion in the cellulose matrix corresponds to bright pixels with respect to black pixels, which represent the non-cellulose portion. The CF is expressed as the ratio of bright pixels (n_C_) to total pixels (bright pixels (n_C_) + black pixels (n_B_)) in terms of percentage in the selected ROI, as reported by Eq. (2)^63^.

2 $$CF= \frac{{n}_{C}}{{n}_{C}+{n}_{B}}$$

Quantitative image analysis is performed by using Fiji software^73^.

### Colorimetric maps for flocs classification

A classification of bioflocs is made by realizing colorimetric maps of the different sets of structural parameters. Precisely, each value is associated to a different colour and three categories are extrapolated depending on the amount (low, medium, high) and structure (unassembled, speckled, assembled) of cellulose present within the bioflocs.

### Microfluidic platform for bioflocs assembly

The microfluidic platform is designed and developed by replica molding of polydimethylsiloxane (PDMS; Sylgard 184; Mascherpa), from a poly (methyl methacrylate) (PMMA, Goodfellow) slab. The microfluidic PMMA master mold is designed by AutoCAD and then carved by micromilling machine (Minithech CNC Mini-Mill). The PMMA layer acts as a mold for the replica molding in Polydimethylsiloxane (PDMS) with a polymer/curing agent ratio of 10:1 (w/w). The chip is characterized by a double layer of PDMS with a sandwiched polycarbonate membrane featuring a porosity of 0.22 µm that is able to separate the culture from the flow. The lower layer is characterized by a central circular, square or triangular chamber with a characteristic length of 7 mm acting as an assembling chamber. The upper PDMS layer has a central microchannel (1.2 mm wide × 50 mm long × 0.6 mm high) and a central circular chamber (12 mm diameter × 0.6 mm high). The PDMS pre-polymer is poured onto each PMMA mold, degassed to remove the air bubbles in the mixture and incubated for 60 min at 80 °C. Once the PDMS is fully cured, it is detached from the mold and finished at the edges with a scalpel. The inlets and outlets of both layers are punched using a 1.5 mm biopsy punch. The circular chamber of the bottom layer is punched using a 5 mm hole punch. Once the PDMS molds of both layers have been obtained, the system is assembled by means of adhesion promoted by oxygen plasma treatment. Firstly, between the two layers, over the assembling chamber, the polycarbonate porous membrane (0.2 μm diameter of pores; Merck Millipore) with a side equal to 15 mm is irreversibly bonded to the lower PDMS layer using a solution based on 5% Aminopropyltriethoxysilane (APTES), an amino silane frequently used in the silanization and functionalization process of surfaces^74^. In detail, the polycarbonate porous membrane is bonded to the lower layer using the revisited method reported by Aran et al.^75^. Briefly, a commercial solution of APTES is diluted in water to 5% by volume and placed at 80 °C on a hot plate for 20 min. The polycarbonate (PC) porous membrane and the lower PDMS layer are oxygen-treated for 1 min at 50 W. After oxygen activation, the two parts are placed in contact and then APTES solution is dropped on the polycarbonate membrane accommodated on PDMS and incubated at 80 °C for 5 min. To bond the upper PDMS layer in a sandwich structure, both upper and lower PDMS layers are oxygen activated, brought in contact and pressed together. Then, the whole setup is incubated at 80 °C overnight to achieve irreversible bonding of the two PDMS layers. Before bacteria culture, the microfluidic device, tubes and connectors are sterilized by autoclaving at 121 °C for 20 min. A peristaltic pump (Cole Parmer) is used to set a flow rate of 200 μL/min in order to deliver appropriate nutrition to the bacteria culture. The apical part of the chip is connected to the flow by the peristaltic pump while in the basal part, the flocs are loaded inside the culture chamber (circular, square or triangular). The chip is temporarily closed by means of a slide holder (24 × 60 mm) and clamps to start the culture, then reopened to collect the sample and analyse it.

### Confocal microscopy

The BC produced by SCOBY bioflocs is stained with calcofluor white (0.002%). Precisely, the dye is diluted within the growth medium during the dynamic culture inside the microfluidic device. Then, images of the assembled bioflocs cellulose are acquired with a confocal microscope (Zeiss, LSM700) (63×) with laser at λ = 405 nm without removing the samples from the device. Cellulose fraction and mean fluorescence intensity are evaluated by using Fiji with the command “measure”.

### Statistical analysis

Data are presented as mean values and standard deviation. Statistical significance between sample populations is evaluated by using the online non-parametric Kruskal–Wallis test followed by post-hoc Dunn's test for multiple comparisons when data are not normally distributed; otherwise, an unpaired Two-Sample t-test is performed. The normality of data is checked by the Shapiro–Wilk test (*p*-values < 0.05 indicates non-normal distribution). Differences are considered statistically significant for *p*-values < 0.05. All the experiments are conducted in triplicate. Spearmans’s correlation coefficient between the ultrastructural and optical parameters (monotonic relationship) is measured in Origin.

### Supplementary Information

Supplementary Figures.

## Data Availability

The datasets generated during and/or analysed during the current study are available from the corresponding authors upon reasonable request.

## References

[1] Urbina L, Corcuera MÁ, Gabilondo N, Eceiza A, Retegi A (2021). A review of bacterial cellulose: Sustainable production from agricultural waste and applications in various fields. Cellulose.

[2] Williams WS, Cannon RE (1989). Alternative environmental roles for cellulose produced by *Acetobacter xylinum*. Appl. Environ. Microbiol..

[3] Rahman SSA (2021). Production of bacterial cellulose using *Gluconacetobacter kombuchae* immobilized on *Luffa aegyptiaca* support. Sci. Rep..

[4] *Comparison of Bacterial Cellulose Production among Different Strains and Fermented Media*.

[5] Gregory DA (2021). Bacterial cellulose: A smart biomaterial with diverse applications. Mater. Sci. Eng. R Rep..

[6] El-Gendi H, Taha TH, Ray JB, Saleh AK (2022). Recent advances in bacterial cellulose: A low-cost effective production media, optimization strategies and applications. Cellulose.

[7] Choi SM, Rao KM, Zo SM, Shin EJ, Han SS (2022). Bacterial cellulose and its applications. Polymers (Basel).

[8] Lupașcu RE (2022). An overview regarding microbial aspects of production and applications of bacterial cellulose. Materials (Basel).

[9] Portela R, Leal CR, Almeida PL, Sobral RG (2019). Bacterial cellulose: A versatile biopolymer for wound dressing applications. Microb. Biotechnol..

[10] Bacakova L (2019). Versatile application of nanocellulose: From industry to skin tissue engineering and wound healing. Nanomaterials (Basel).

[11] Swingler S (2021). Recent advances and applications of bacterial cellulose in biomedicine. Polymers (Basel).

[12] Salimi S, Sotudeh-Gharebagh R, Zarghami R, Chan SY, Yuen KH (2019). Production of nanocellulose and its applications in drug delivery: A critical review. ACS Sustain. Chem. Eng..

[13] Unal S, Gunduz O, Uzun M, Ahmed S, Ali W (2020). Tissue engineering applications of bacterial cellulose based nanofibers. Green Nanomaterials.

[14] Moniri M (2017). Production and status of bacterial cellulose in biomedical engineering. Nanomaterials (Basel).

[15] Castro C (2011). Structural characterization of bacterial cellulose produced by *Gluconacetobacter swingsii* sp. from Colombian agroindustrial wastes. Carbohydr. Polym..

[16] Al-Shamary E, Esmaeel AKA-D (2013). Influence of fermentation condition and alkali treatment on the porosity and thickness of bacterial cellulose membranes. Tojsat.

[17] Andritsou V (2018). Synthesis and characterization of bacterial cellulose from citrus-based sustainable resources. ACS Omega.

[18] Molina-Ramírez C (2017). Effect of different carbon sources on bacterial nanocellulose production and structure using the low pH resistant strain *Komagataeibacter medellinensis*. Materials (Basel).

[19] Feng X (2024). Production and characterization of bacterial cellulose from kombucha-fermented soy whey. Food Prod. Process. Nutr..

[20] Fatima A, Ortiz-Albo P, Neves LA, Nascimento FX, Crespo JG (2023). Biosynthesis and characterization of bacterial cellulose membranes presenting relevant characteristics for air/gas filtration. J. Memb. Sci..

[21] Troncoso OP, Torres FG (2020). Bacterial cellulose-graphene based nanocomposites. Int. J. Mol. Sci..

[22] Torres FG, Arroyo JJ, Troncoso OP (2019). Bacterial cellulose nanocomposites: An all-nano type of material. Mater. Sci. Eng. C Mater. Biol. Appl..

[23] McCarthy RR, Ullah MW, Booth P, Pei E, Yang G (2019). The use of bacterial polysaccharides in bioprinting. Biotechnol. Adv..

[24] Tai C (2019). Use of anionic polysaccharides in the development of 3D bioprinting technology. Appl. Sci. (Basel).

[25] Schaffner M, Rühs PA, Coulter F, Kilcher S, Studart AR (2017). 3D printing of bacteria into functional complex materials. Sci. Adv..

[26] Athukoralalage SS, Balu R, Dutta NK, Roy Choudhury N (2019). 3D bioprinted nanocellulose-based hydrogels for tissue engineering applications: A brief review. Polymers (Basel).

[27] Markstedt K (2015). 3D bioprinting human chondrocytes with nanocellulose-alginate bioink for cartilage tissue engineering applications. Biomacromolecules.

[28] Huang L (2019). Bacterial cellulose nanofibers promote stress and fidelity of 3D-printed silk based hydrogel scaffold with hierarchical pores. Carbohydr. Polym..

[29] Müller M, Öztürk E, Arlov Ø, Gatenholm P, Zenobi-Wong M (2017). Alginate sulfate-nanocellulose bioinks for cartilage bioprinting applications. Ann. Biomed. Eng..

[30] Wu Z (2021). Biocompatibility evaluation of a 3D-bioprinted alginate-GelMA-bacteria nanocellulose (BNC) scaffold laden with oriented-growth RSC96 cells. Mater. Sci. Eng. C Mater. Biol. Appl..

[31] Pillai MM (2021). Symbiotic culture of nanocellulose pellicle: A potential matrix for 3D bioprinting. Mater. Sci. Eng. C Mater. Biol. Appl..

[32] Coelho RMD, de Almeida AL, Amaral RQGD, da Mota RN, de Sousa PHM (2020). Kombucha: Review. Int. J. Gastron. Food Sci..

[33] Kitwetcharoen H (2023). Kombucha healthy drink—recent advances in production, chemical composition and health benefits. Fermentation.

[34] Jayabalan R, Malbaša RV, Lončar ES, Vitas JS, Sathishkumar M (2014). A review on kombucha tea—microbiology, composition, fermentation, beneficial effects, toxicity, and tea fungus. Compr. Rev. Food Sci. Food Saf..

[35] Ramírez Tapias YA, Peltzer MA, Delgado JF, Salvay AG (2020). Kombucha tea by-product as source of novel materials: Formulation and characterization of films. Food Bioproc. Tech..

[36] Betlej I, Salerno-Kochan R, Krajewski KJ, Zawadzki J, Boruszewski P (2020). The influence of culture medium components on the physical and mechanical properties of cellulose synthesized by kombucha microorganisms. Bioresources.

[37] Villarreal-Soto SA (2019). Impact of fermentation conditions on the production of bioactive compounds with anticancer, anti-inflammatory and antioxidant properties in kombucha tea extracts. Process Biochem..

[38] Tsilo PH, Basson AK, Ntombela ZG, Maliehe TS, Pullabhotla RVSR (2021). Isolation and optimization of culture conditions of a bioflocculant-producing fungi from Kombucha tea SCOBY. Microbiol. Res. (Pavia).

[39] Oyewole OA (2023). Production and characterization of a bioflocculant produced by microorganisms isolated from earthen pond sludge. Bioresour. Technol. Rep..

[40] Kurniawan SB (2020). Challenges and opportunities of biocoagulant/bioflocculant application for drinking water and wastewater treatment and its potential for sludge recovery. Int. J. Environ. Res. Public Health.

[41] Mohammed JN, Wan Dagang WRZ (2019). Implications for industrial application of bioflocculant demand alternatives to conventional media: Waste as a substitute. Water Sci. Technol..

[42] Giri SS, Ryu E, Park SC (2019). Characterization of the antioxidant and anti-inflammatory properties of a polysaccharide-based bioflocculant from Bacillus subtilis F9. Microb. Pathog..

[43] Thu TTM, Moreira RA, Weber SAL, Poma AB (2022). Molecular insight into the self-assembly process of cellulose Iβ microfibril. Int. J. Mol. Sci..

[44] Kim J, Park H-D, Chung S (2012). Microfluidic approaches to bacterial biofilm formation. Molecules.

[45] Tsuboi M (1957). Infrared spectrum and crystal structure of cellulose. J. Polym. Sci..

[46] Li J (2019). Production of high crystallinity type-I cellulose from Komagataeibacter hansenii JR-02 isolated from Kombucha tea. Biotechnol. Appl. Biochem..

[47] Amarasekara AS, Wang D, Grady TL (2020). A comparison of kombucha SCOBY bacterial cellulose purification methods. SN Appl. Sci..

[48] Peretz R, Mamane H, Sterenzon E, Gerchman Y (2019). Rapid quantification of cellulose nanocrystals by Calcofluor White fluorescence staining. Cellulose.

[49] *Nanocellulose-Based Inks for 3D Bioprinting: Key Aspects in ResearchDevelopment and Challenging Perspectives in Applications-AMini Review*. 10.3390/bioengineering7020040 PMC7355978 32365578

[50] Williams D, Thayer P, Martinez H, Gatenholm E, Khademhosseini A (2018). A perspective on the physical, mechanical and biological specifications of bioinks and the development of functional tissues in 3D bioprinting. Bioprinting.

[51] Ben Rebah F, Mnif W, Siddeeg MS (2018). Microbial flocculants as an alternative to synthetic polymers for wastewater treatment: A review. Symmetry (Basel).

[52] Olivero E (2023). Gradient porous structures of mycelium: A quantitative structure-mechanical property analysis. Res. Square.

[53] Di Natale C (2022). Engineered bacterial cellulose nanostructured matrix for incubation and release of drug-loaded oil in water nanoemulsion. Front. Bioeng. Biotechnol..

[54] Brown RM, Millard AC, Campagnola PJ (2003). Macromolecular structure of cellulose studied by second-harmonic generation imaging microscopy. Opt. Lett..

[55] Nadiarnykh O, Lacomb RB, Campagnola PJ, Mohler WA (2007). Coherent and incoherent SHG in fibrillar cellulose matrices. Opt. Express.

[56] Vielreicher M (2018). Bacterial nanocellulose stimulates mesenchymal stem cell expansion and formation of stable collagen-I networks as a novel biomaterial in tissue engineering. Sci. Rep..

[57] Sharma C, Bhardwaj NK (2019). Biotransformation of fermented black tea into bacterial nanocellulose via symbiotic interplay of microorganisms. Int. J. Biol. Macromol..

[58] Bäckdahl H (2006). Mechanical properties of bacterial cellulose and interactions with smooth muscle cells. Biomaterials.

[59] Tang W, Jia S, Jia Y, Yang H (2010). The influence of fermentation conditions and post-treatment methods on porosity of bacterial cellulose membrane. World J. Microbiol. Biotechnol..

[60] Sharma C, Bhardwaj NK, Pathak P (2021). Static intermittent fed-batch production of bacterial nanocellulose from black tea and its modification using chitosan to develop antibacterial green packaging material. J. Clean. Prod..

[61] Hornung M, Ludwig M, Gerrard AM, Schmauder H-P (2006). Optimizing the production of bacterial cellulose in surface culture: Evaluation of substrate mass transfer influences on the bioreaction (part 1). Eng. Life Sci..

[62] Vasconcelos NF (2020). Oxidized bacterial cellulose membrane as support for enzyme immobilization: Properties and morphological features. Cellulose.

[63] De Gregorio V (2020). Intestine-on-chip device increases ECM remodeling inducing faster epithelial cell differentiation. Biotechnol. Bioeng..

[64] De Gregorio V (2022). Immunoresponsive microbiota-gut-on-chip reproduces barrier dysfunction, stromal reshaping and probiotics translocation under inflammation. Biomaterials.

[65] Zhong C (2020). Industrial-scale production and applications of bacterial cellulose. Front. Bioeng. Biotechnol..

[66] Iguchi M, Yamanaka S, Budhiono A (2000). Bacterial cellulose—A masterpiece of nature's arts. J. Mater. Sci..

[67] Park A, Jeong H-H, Lee J, Kim KP, Lee C-S (2011). Effect of shear stress on the formation of bacterial biofilm in a microfluidic channel. Biochip J..

[68] Chao Y, Sugano Y, Shoda M (2001). Bacterial cellulose production under oxygen-enriched air at different fructose concentrations in a 50-liter, internal-loop airlift reactor. Appl. Microbiol. Biotechnol..

[69] Lombardo D, Calandra P, Pasqua L, Magazù S (2020). Self-assembly of organic nanomaterials and biomaterials: The bottom-up approach for functional nanostructures formation and advanced applications. Materials (Basel).

[70] Caro-Astorga J, Walker KT, Herrera N, Lee K-Y, Ellis T (2021). Bacterial cellulose spheroids as building blocks for 3D and patterned living materials and for regeneration. Nat. Commun..

[71] Indurkar A, Choudhary R, Rubenis K, Locs J (2021). Advances in sintering techniques for calcium phosphates ceramics. Materials (Basel).

[72] Hotaling NA, Bharti K, Kriel H, Simon CG (2015). DiameterJ: A validated open source nanofiber diameter measurement tool. Biomaterials.

[73] Schindelin J (2012). Fiji: An open-source platform for biological-image analysis. Nat. Methods.

[74] Agostini M, Greco G, Cecchini M (2019). Polydimethylsiloxane (PDMS) irreversible bonding to untreated plastics and metals for microfluidics applications. APL Mater..

[75] Aran K, Sasso LA, Kamdar N, Zahn JD (2010). Irreversible, direct bonding of nanoporous polymer membranes to PDMS or glass microdevices. Lab. Chip..

