# Supplementary material for: Exploring the evolution of bacterial cellulose precursors and their potential use as cellulose-based building blocks
Source: Sci Rep. 2024 May 21;14:11613. doi: 10.1038/s41598-024-62462-9 (PMC11109180; doi:10.1038/s41598-024-62462-9)
Supplement: Supplementary file 1 — Supplementary Figures. [file 41598_2024_62462_MOESM1_ESM.pdf]

Supporting Information

Exploring the evolution of bacterial cellulose precursors and their potential use as cellulose-based building blocks

Francesca Mauro[1,2], Brunella Corrado[3], Vincenza De Gregorio[4*], Elena Lagreca[2], Concetta di Natale[1], Raffaele Vecchione[2*] and Paolo Antonio Netti[1,2,3].

[1]Department of Chemical Materials, Industrial Production Engineering, University of Naples Federico II, Naples, Italy

[2]Istituto Italiano di Tecnologia, Naples, Italy

[3]Interdisciplinary Research Centre on Biomaterials, University of Naples Federico II, Naples, Italy

[4]Department of Biology, University of Naples Federico II

*corresponding authors: raffaele.vecchione@iit.it, vincenzadegregorio@unina.it

Keywords: Bacterial cellulose, Kombucha, biomaterials, SEM, SHG

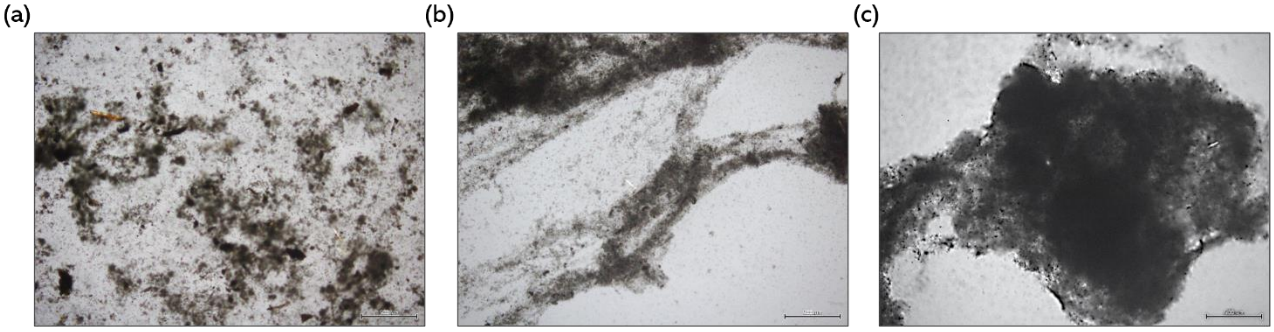

Figure S1. SCOBY bioflocs microscopic images. Optical images in brightfield (Olympus) (4×) illustrating SCOBY bioflocs obtained from the bottom (a), middle (b), and top (c) vertical positions of the culture liquid column within the tube. The scale bar is 500 µm.

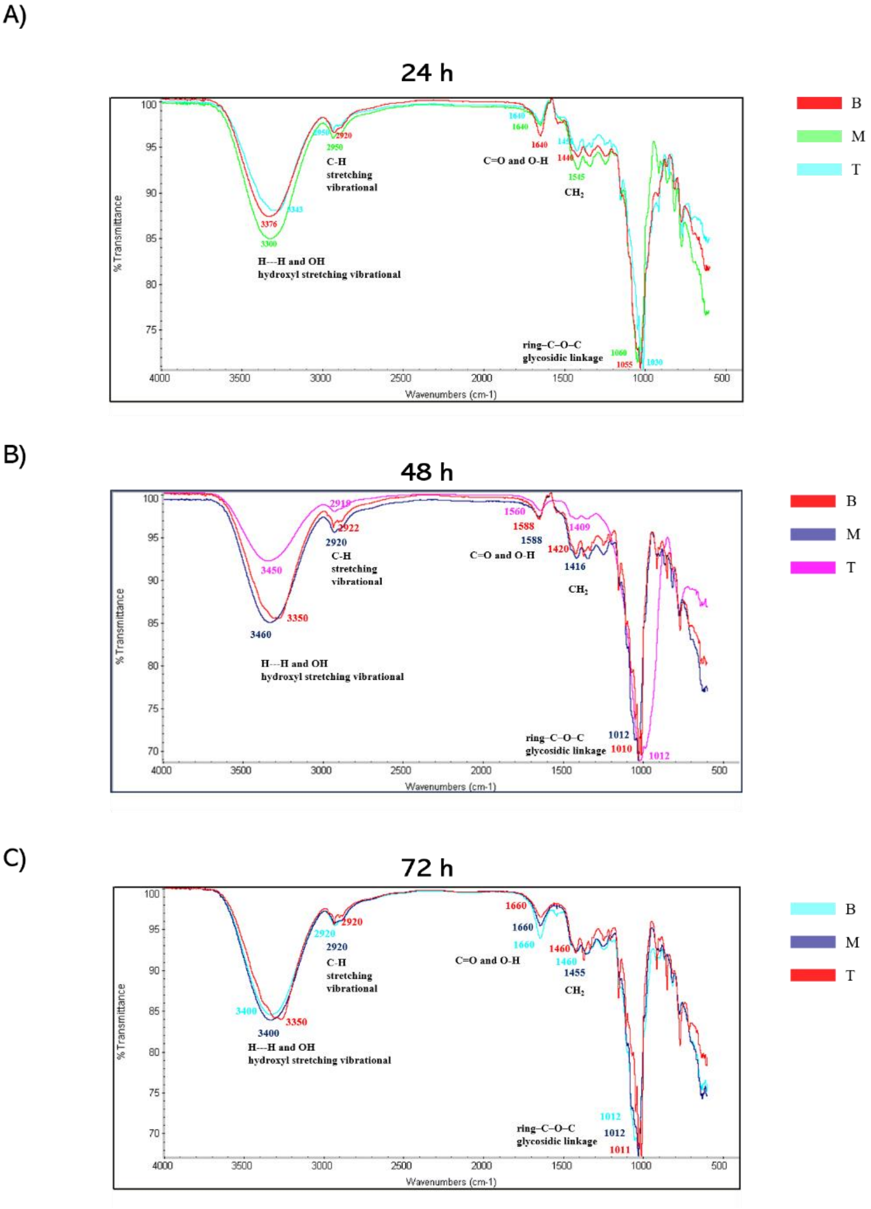

Figure S2. IR analysis. IR analyses of SCOBY bioflocs obtained from the bottom (B), middle (M), and top (T) vertical positions of the culture broth at 24 h, 48 h, and 72 h.

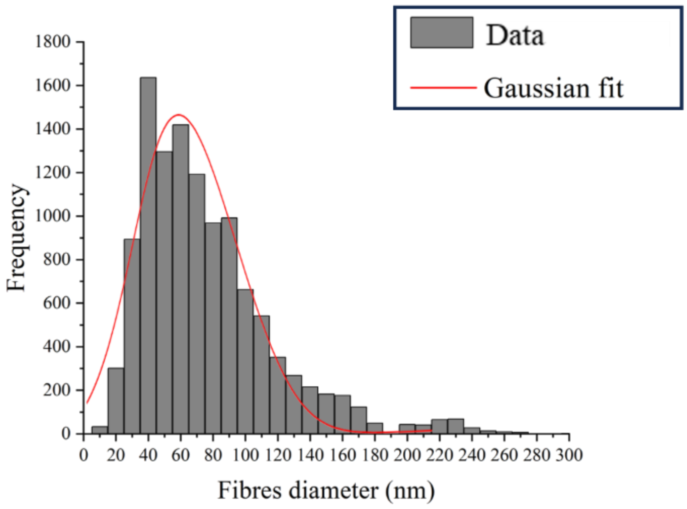

Figure S3. SCOBY bioflocs fibres' diamaeter. Histogram illustrating the diameter of cellulose fibres in SCOBY bioflocs. The mean value of 60 nm was derived through Gaussian fitting of the distribution in Origin (red line).

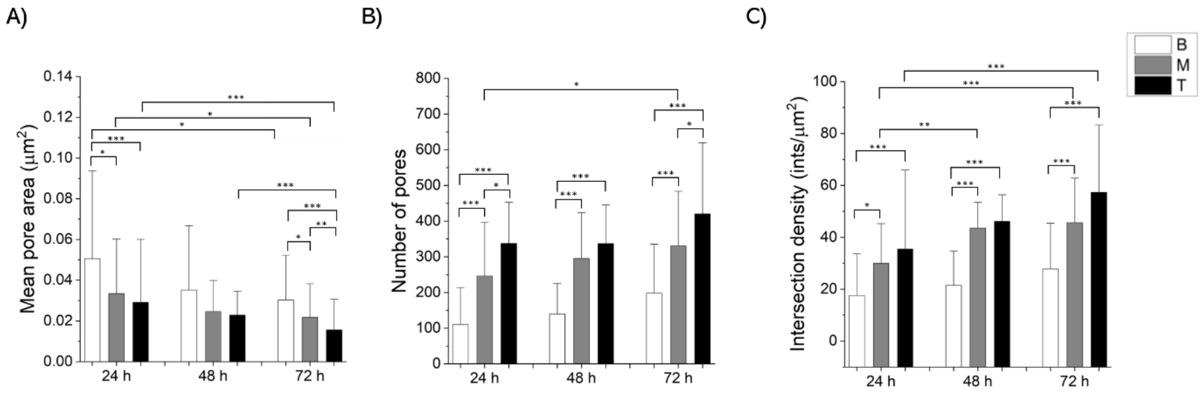

Figure S4. Analysis of SCOBY Bioflocs Network. A) Evaluation of mean pore area, B) number of pores and C) intersection density of bioflocs cellulose network. The bioflocs were collected from bottom (B), middle (M), and top (T) vertical positions of the culture broth at 24 h, 48 h, and 72 h. Statistical significance is assessed through the Kruskal-Wallis test (***$p < 0.005$, *$p < 0.05$, not significant when not shown, $n \geq 12$). Data are presented as mean values and standard deviation.

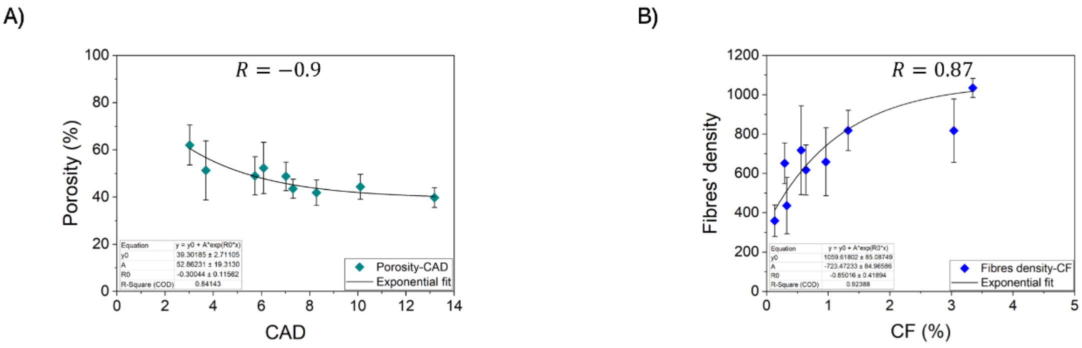

Figure S5. SEM-SHG parameters correlation. Graphs representing the relationship between porosity and CAD (A), as well as between fibre amount and CF (B). Data points represented their mean values, while error bars are the standard deviations. Exponential fitting was applied to characterize the relation between SEM/SHG parameters. Spearman's correlation coefficient (R) is presented in each graph, revealing a negative correlation between porosity and CAD, and a positive correlation between cellulose fibre amount and CF.

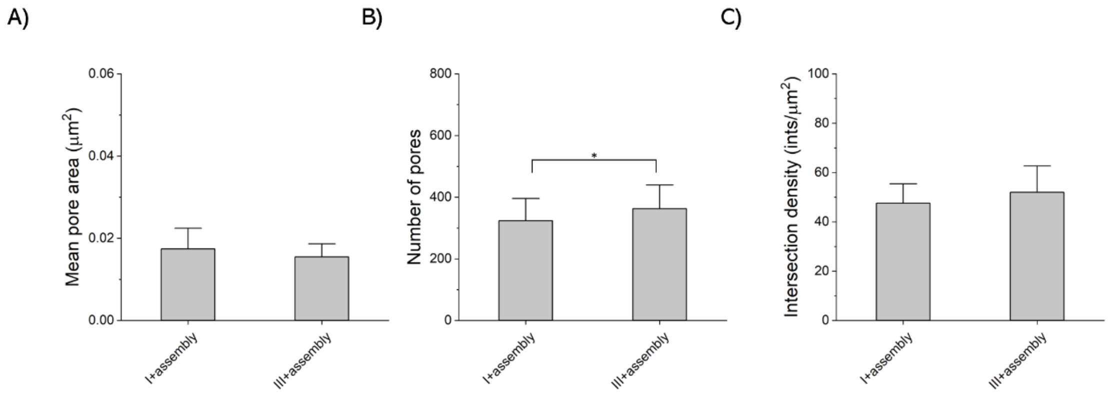

Figure S6. Structural analysis of cellulose sheet network derived from assembled bioflocs. Results of A) Mean pore area, B) number of pores and C) fibre intersection density. These assessments were conducted on assembled class I and class III SCOBY bioflocs after 24 h of culture within the microfluidic device. Statistical significance is assessed through a Two-sample t-test (*$p < 0.05$, not significant when not shown, $n \geq 10$). Data are presented as mean values and standard deviation.

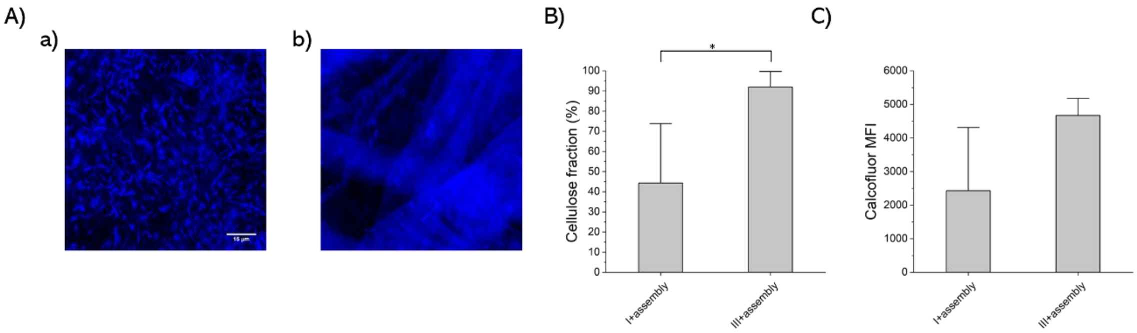

Figure S7. Confocal microscopy assessment of cellulose sheet derived from the assembled bioflocs. A) Confocal microscopy images of assembled class I a) and class III b) SCOBY bioflocs after 24 h of culture within the microfluidic device, stained with calcofluor white (0.002 %). Results of (B) cellulose fraction and (C) mean fluorescence intensity (MFI). Statistical significance is assessed Two-sample t-test (*p < 0.05, not significant when not shown, n ≥ 10). Data are presented as mean values and standard deviation.
